# Hypoparathyroidism after total thyroidectomy in patients with previous gastric bypass

**DOI:** 10.1007/s00423-016-1517-x

**Published:** 2016-10-26

**Authors:** Raoul A. Droeser, Johan Ottosson, Andreas Muth, Hella Hultin, Karin Lindwall-Åhlander, Anders Bergenfelz, Martin Almquist

**Affiliations:** 1 Sten Tibblin Fellow, Dept. of Surgery, Skane University Hospital, Lund, Sweden; 2 Dept. of Surgery, Faculty of Medicine and Health, Örebro University, Örebro, Sweden; 3 Dept. of Surgery, Institute of Clinical Sciences, Sahlgrenska Academy, University of Gothenburg, Gothenburg, Sweden; 4 Dept. of Surgery, Akademiska Hospital, Uppsala, Sweden; 5 Dept. of Surgery, Gävle County Hospital, Gävle, Sweden; 6 Dept. of Surgery, Skane University Hospital, S-221 85 Lund, Sweden; 7 Lund University, S-221 85 Lund, Sweden

**Keywords:** Total thyroidectomy, Gastric bypass, Postoperative hypoparathyroidism, Cohort study

## Abstract

**Purpose:**

Case reports suggest that patients with previous gastric bypass have an increased risk of severe hypocalcemia after total thyroidectomy, but there are no population-based studies. The prevalence of gastric bypass before thyroidectomy and the risk of hypocalcemia after thyroidectomy in patients with previous gastric bypass were investigated.

**Methods:**

By cross-linking The Scandinavian Quality Registry for Thyroid, Parathyroid and Adrenal Surgery with the Scandinavian Obesity Surgery Registry patients operated with total thyroidectomy without concurrent or previous surgery for hyperparathyroidism were identified and grouped according to previous gastric bypass. The risk of treatment with intravenous calcium during hospital stay, and with oral calcium and vitamin D at 6 weeks and 6 months postoperatively was calculated by using multiple logistic regression in the overall cohort and in a 1:1 nested case-control analysis.

**Results:**

We identified 6115 patients treated with total thyroidectomy. Out of these, 25 (0.4 %) had undergone previous gastric bypass surgery. In logistic regression, previous gastric bypass was not associated with treatment with i.v. calcium (OR 2.05, 95 % CI 0.48–8.74), or calcium and/or vitamin D at 6 weeks (1.14 (0.39–3.35), 1.31 (0.39–4.42)) or 6 months after total thyroidectomy (1.71 (0.40–7.32), 2.28 (0.53–9.75)). In the nested case-control analysis, rates of treatment for hypocalcemia were similar in patients with and without previous gastric bypass.

**Conclusion:**

Previous gastric bypass surgery was infrequent in patients undergoing total thyroidectomy and was not associated with an increased risk of postoperative hypocalcemia.

**Electronic supplementary material:**

The online version of this article (doi:10.1007/s00423-016-1517-x) contains supplementary material, which is available to authorized users.

## Introduction

Several case reports indicate that previous bariatric surgery for morbid obesity constitutes a risk factor for severe hypocalcemia after total thyroidectomy (TT) [1–3]. Gastric bypass surgery (GBP) is the most commonly performed procedure for morbid obesity, and due to the worldwide obesity epidemic, the prevalence of previous GBP is increasing [4]. Hypocalcemia is the most common complication of TT [5] [6] and is caused by intraoperative injury to the parathyroid glands or their blood supply [7]. Low level vitamin D can also aggravate postoperative hypocalcemia [8]. Patients with GBP are at risk of developing nutritional deficiencies [9] [10]. In GBP, the absorption of calcium and vitamin D is diminished, possibly due to the exclusion of the duodenum and proximal jejunum, where active vitamin D-associated calcium uptake occurs [11]. Accordingly, patients with GBP have lower levels of vitamin D (25OHD) than the general population [12]. However, it has been shown that 25OHD levels in GBP patients are equally low before and after GBP [13]. Low levels of 25OHD also persist at long-term follow up after GBP, together with the increased levels of parathyroid hormone (PTH), decreased bone mineral density, and increased risk of fractures [12–14]. This has led to the recommendation of calcium and vitamin D supplementation after GBP [15]. Thus, patients with previous GBP could be at increased risk of hypocalcemia after TT.

Hypocalcemia after TT can cause unpleasant symptoms, leading to increased costs for sick leave, follow-up visits, and readmission [16] [17]. Therefore, identifying risk factors for hypocalcemia is important. Patients with increased risk for this complication could benefit from a longer hospital stay, increased surveillance, and/or preoperative treatment with calcium and/or vitamin D.

Apart from case reports [1–3], there has been no population-based study investigating hypocalcemia after TT in relation to GBP. Thus, we aimed to investigate the frequency and severity of postoperative hypocalcemia in patients with previous GBP undergoing TT, and whether GBP increases the risk for hypocalcemia after TT, using data from nationwide, clinical registers.

## Material and methods

### Data sources

#### SQRTPA

The Scandinavian Quality Registry for Thyroid, Parathyroid and Adrenal Surgery (SQRTPA, www.thyroid-parathyroidsurgery.com) started in 2004 and is recognized by the Swedish National Board for Health and Social Welfare as the national quality registry for endocrine surgical procedures in Sweden. Currently, 35 Swedish units report to the registry, covering 88 % of all procedures during the study period. Coverage is assessed by calculating the proportion of patients registered in the SQRTPA in relation to those registered in the Inpatient Register of the Swedish National Board of Health and Welfare. The quality of data for the registered patients is checked by external audit, comparing registered data to hospital medical records. The audit has demonstrated good data quality, with an error rate of <5 % [18, 19].

#### SOReg

The Swedish Obesity Registry, SOReg, started in May 2007. All Swedish bariatric units are affiliated with this register. The national coverage is 97 % and regular data quality evaluations have shown that >98 % of data are correct [20].

### Study cohort

Patients operated with TT between 1 January 2004 and 31 December 2015 were identified in the SQRTPA. Patients with concurrent or previous surgery for hyperparathyroidism were excluded, as were patients with missing information on postoperative hypocalcemia. By cross-linking with the SOReg, information on GBP was obtained.

### Ethical considerations

This study was approved by the research ethics committee at Lund University, DNR 2015/543 and 2016/83.

### Data

Endpoint data concerning postoperative oral and intravenous (i.v.) calcium and vitamin D treatment during hospital stay, at discharge, and at 6 weeks and 6 months postoperatively were extracted from the SQRTPA. Further, data on age, sex, indication for thyroidectomy, and lymph node dissection were retrieved.

### Statistical analysis

The risk of hypocalcemia after TT in patients with and without previous GBP was investigated both in the whole cohort and in a 1:1 nested case-control subset. In the nested case-control group, patients with TT with previous GBP were randomly matched to patients without previous GBP, on the factors age, gender, lymph node dissection or not, and indication for surgery.

Means and standard deviations (SD) were calculated for continuous variables except for age. Age is reported as median and range. Numbers and column percentages were calculated for categorical variables.

In the whole cohort, risk for treatment with calcium and vitamin D at different times of follow-up, in patients with and without GBP, was investigated with uni- and multivariate logistic regression, adjusting for possible confounding variables: age, gender, indication for surgery, and lymph node dissection. In the nested case-control subset, ANOVA, chi-square, or Fisher’s exact tests were used to determine the association of primary endpoints with previous GBP. All analyses were performed with STATA 13 (StataCorp LP, College Station, TX, USA). A *p* value of less than 0.05 was considered statistically significant.

This study was performed and reported according to the Strengthening the Reporting of Observational Studies in Epidemiology statement [21].

## Results

### Baseline patient and procedure characteristics in the overall cohort

There were 7600 patients who underwent TT. Of these, we excluded 217 patients due to prior or concurrent parathyroidectomy. Another seven patients lacked information on treatment for postoperative hypocalcemia. Patients that were operated before 1 May 2007 (start date of the SOReg) were also excluded (*N* = 1261). This left 6115 patients for analysis. By cross-linking with SOReg, 25 patients with GBP before TT were identified, corresponding to a rate of 25/6115 (0.4 %).

The median age in the cohort was 46 (33–59), male to female ratio was 1:4, and the indication for surgery was thyrotoxicosis in half of the patients. Mean (SD) postoperative total calcium was 2.13 (±0.20; Table 1) at day one. Total calcium at 6-week and 6-month follow-up were 2.30 (±0.17) and 2.25 (±0.17), respectively. At 6-week follow-up, 841 (13.8 %) had oral calcium and 554 patients (9.1 %) vitamin D substitution for hypocalcemia. These numbers were 290 (4.7 %) and 214 (3.5 %) at 6-month follow-up. Baseline characteristics are summarized in Table 1.

**Table 1:** Characteristics of patients operated with total thyroidectomy registered in the SQRTPA from 1 May 2007–2015 (*N* = 6115)

	*N* = 6115 (100 %)
Age median (IQR)	46 (33–59)
Sex	
Male	1234 (20.2)
Female	4881 (79.8)
Indication for thyroidectomy	
Recurrent cyst	12 (0.2)
Completion operation	17 (0.3)
Excluding malignancy	457 (7.5)
Malignancy	956 (15.6)
Compression	1586 (25.9)
Thyrotoxicosis	3063 (50.1)
Other	24 (0.4)
Lymphnode dissection	
Yes	1068 (17.5)
No	5047 (82.5)
Gland weight (grams)	74.2 (±94.0)
Oral perioperative calcium treatment	
Yes	2055 (33.6)
No	4060 (66.4)
Intravenous calcium treatment	
Yes	250 (4.1)
No	5865 (95.9)
Peroral calcium treatment	
At discharge (yes/no)	1776 (29.0)/4339 (71.0)
At 6 weeks follow-up (yes/no)	841 (13.8)/4793 (78.4)
At 6 months follow-up (yes/no)	290 (4.7)/5184 (84.8)
Total serum calcium	
Postoperative day 1	2.13 (±0.20)
At 6 weeks follow-up	2.30 (±0.17)
At 6 months follow-up	2.25 (±0.17)
Peroral vitamin D treatment	
At discharge (yes/no)	909 (14.9)/5206 (85.1)
At 6 weeks follow-up (yes/no)	554 (9.1)/5087 (83.2)
At 6 months follow-up (yes/no)	214 (3.5)/5333 (87.2)

*IQR* interquartile range, *GBP* gastric bypass

The number of patients without follow-up information for oral calcium treatment at 6 weeks and 6 months were 481 (7.9 %) and 641 (10.5 %), respectively.

### Patient and procedure characteristics in patients with and without previous GBP

In the whole cohort, there were no statistically significant differences between patients with and without previous GBP regarding age (median, (IQR)) 48 (41–52) vs 46 (33–59); *p* = 0.977), male to female ratio (4/21 vs 1230/4860; *p* = 0.602) and postoperative total calcium (mean, (SD) 2.13 (±0.16) mmol/l vs 2.12 (±0.20) mmol/l; *p* = 0.997). However, there was a significant difference for the indication for thyroidectomy (*p* = 0.015). The most common indication for TT overall was thyreotoxicosis; in patients with previous GBP, it was compression symptoms.

Total serum calcium and rates of calcium and/or vitamin D substitution at 6-week and 6-month follow-up after thyroidectomy did not differ significantly between patients with and without previous GBP in the whole cohort (Table 2).

**Table 2:** Endpoint characteristics of group without and with previous GBP in the overall cohort (*N* = 6115)

	No previous GBP *N* = 6090 (100 %)	Previous GBP *N* = 25 (100 %)	*p* value
Age median (IQR)	46 (33–59)	48 (41–52)	0.977
Sex			
Male	1230 (20.2)	4 (16.0)	0.602
Female	4860 (79.8)	21 (84.0)	
Indication for thyroidectomy			
Recurrent cyst	12 (0.2)	0 (0.0)	0.015
Completion operation	17 (0.3)	0 (0.0)	
Excluding malignancy	454 (7.5)	3 (12.0)	
Malignancy	949 (15.6)	7 (28.0)	
Compression	1575 (25.9)	11 (44.0)	
Thyreotoxicosis	3059 (50.2)	4 (16.0)	
Other	24 (0.4)	0 (0.0)	
Lymphnode dissection			
Yes	1061 (17.4)	7 (28.0)	0.164
No	5029 (82.6)	18 (72.0)	
Gland weight (grams)	74.1 (±94.0)	80.7 (±72.2)	0.748
Oral perioperative calcium treatment			
Yes	2047 (33.6)	8 (32.0)	0.865
No	4043 (66.4)	17 (68.0)	
Intravenous calcium treatment			
Yes	248 (4.1)	2 (8.0)	0.322
No	5842 (95.9)	23 (92.0)	
Peroral calcium treatment			
At discharge (yes/no)	1770 (29.1)/4320 (70.9)	6 (24.0)/19 (76.0)	0.578
At 6 weeks follow-up (yes/no)	837 (13.7)/4773 (78.4)	4 (16.0)/20 (80.0)	0.651
At 6 months follow-up (yes/no)	288 (4.7)/5163 (84.8)	2 (8.0)/21 (84.0)	0.373
Total serum calcium			
Postoperative day 1	2.12 (±0.20)	2.13 (±0.16)	0.997
At 6 weeks follow-up	2.30 (±0.17)	2.27 (±0.13)	0.305
At 6 months follow-up	2.25 (±0.17)	2.26 (±0.10)	0.901
Peroral vitamin D treatment			
At discharge (yes/no)	904 (14.8)/5186 (85.2)	5 (20.0)/20 (80.0)	0.470
At 6 weeks follow-up (yes/no)	551 (9.0)/5066 (83.2)	3 (12.0)/21 (84.0)	0.616
At 6 months follow-up (yes/no)	212 (3.5)/5311 (87.2)	2 (8.0)/22 (88.0)	0.278

*IQR* interquartile range, *GBP* gastric bypass

### Logistic regression analysis of hypocalcemia, vitamin D and calcium treatment at discharge and follow-up in the whole cohort

In the multivariate logistic regression analysis including age, gender, lymph node dissection, and indication for surgery, previous gastric bypass was not associated with in hospital treatment with i.v. or oral calcium (2.07 (0.48–9.07); 0.89 (0.37–2.11)), oral calcium, and/or vitamin D at discharge (0.75 (0.29–1.91); 1.37 (0.50–3.76)), at 6 weeks (1.11 (0.37–3.31); 1.25 (0.36–4.27)), or 6 months postoperatively (1.45 (0.33–6.36); 2.01 (0.46–8.77); Tables 3 and 4).

**Table 3:** Uni- and multivariate logistic regression analysis for calcium treatment after total thyroidectomy

Factor	In hospital i.v. calcium		Per oral calcium at discharge		Per oral calcium at 6 weeks		Per oral calcium at 6 months	
	OR (95 % CI)	OR^a^ (95 % CI)	OR (95 % CI)	OR^a^ (95 % CI)	OR (95 % CI)	OR^a^ (95 % CI)	OR (95 % CI)	OR^a^ (95 % CI)
Age	0.98 (0.97–0.98)	0.98 (0.97–0.99)	1.00 (1.01–1.42)	0.99 (0.99–0.99)	1.00 (0.99–1.00)	1.00 (0.99–1.00)	1.00 (0.99–1.00)	0.99 (0.99–1.00)
Lymph node dissection	1.67 (1.24–2.23)	2.04 (1.48–2.82)	2.20 (1.92–2.52)	2.49 (2.16–2.89)	2.03 (1.72–2.40)	2.26 (1.88–2.70)	2.85 (2.22–3.66)	2.97 (2.27–3.88)
Indication for surgery	1.09 (1.03–1.16)	1.06 (0.99–1.14)	1.05 (1.02–1.07)	1.07 (1.04–1.10)	1.03 (1.00–1.07)	1.07 (1.02–1.11)	0.97 (0.92–1.02)	1.01 (0.94–1.08)
Gender	0.84 (0.60–1.17)	0.85 (0.60–1.19)	1.02 (0.89–1.17)	0.94 (0.81–1.08)	1.06 (0.89–1.27)	0.97 (0.81–1.16)	1.10 (0.83–1.47)	0.94 (0.70–1.26)
Previous GBP	2.05 (0.48–8.74)	2.07 (0.48–9.07)	0.77 (0.31–1.93)	0.75 (0.29–1.91)	1.14 (0.39–3.35)	1.11 (0.37–3.31)	1.71 (0.40–7.32)	1.45 (0.33–6.36)

*GBP* gastric bypass

^a^Adjusted for age, lymph node dissection, indication for surgery, gender, and previous GBP

**Table 4:** Uni- and multivariate logistic regression analysis for vitamin D treatment after total thyroidectomy

Factor	Per oral vitamin D at discharge		Per oral vitamin D at 6 weeks		Per oral vitamin D at 6 months	
	OR (95 % CI)	OR^a^ (95 % CI)	OR (95 % CI)	OR^a^ (95 % CI)	OR (95 % CI)	OR^a^ (95 % CI)
Age	0.99 (0.98–0.99)	0.99 (0.98–0.99)	0.99 (0.99–1.00)	0.99 (0.99–1.00)	1.00 (0.99–1.00)	0.99 (0.99–1.00)
Lymph node dissection	2.17 (1.84–2.55)	2.52 (2.12–3.01)	2.00 (1.64–2.44)	2.29 (1.85–2.84)	2.72 (2.04–3.62)	2.85 (2.09–3.88)
Indication for surgery	1.04 (1.01–1.08)	1.05 (1.01–1.10)	1.04 (1.00–1.08)	1.05 (1.00–1.11)	0.98 (0.92–1.04)	1.02 (0.94–1.10)
Gender	0.91 (0.76–1.09)	0.84 (0.70–1.01)	0.92 (0.73–1.15)	0.85 (0.67–1.07)	1.12 (0.80–1.55)	0.95 (0.68–1.34)
Previous GBP	1.43 (0.54–3.83)	1.37 (0.50–3.76)	1.31 (0.39–4.42)	1.25 (0.36–4.27)	2.28 (0.53–9.74)	2.01 (0.46–8.77)

*GBP* gastric bypass

^a^Adjusted for age, lymph node dissection, indication for surgery, gender, and previous GBP

### Endpoints in patients with and without previous GBP in the nested case-control group

For the matched nested case-control analysis, the 25 patients with GBP before TT were successfully matched to an equal number of controls. Mean postoperative total calcium on day one was 2.13 (±0.16) mmol/l in patients with previous GBP, vs 2.16 (±0.15; *p* = 0.450) mmol/l in patients without. Mean total serum calcium and rates of calcium and/or vitamin D substitution at 6-week and 6-month follow-up after thyroidectomy did not differ between patients with and without previous GBP (Table 5).

**Table 5:** Results of 1:1 nested case-control analysis

	No previous GBP *N* = 25 (100 %)	Previous GBP *N* = 25 (100 %)	*p* value
Age median (IQR)	48 (41–52)	48 (41–52)	1.000
Sex			
Male	4 (16.0)	4 (16.0)	1.000
Female	21 (84.0)	21 (84.0)	
Indication for thyroidectomy			
Recurrent cyst	0 (0.0)	0 (0.0)	1.000
Completion operation	0 (0.0)	0 (0.0)	
Excluding malignancy	3 (12.0)	3 (12.0)	
Malignancy	7 (28.0)	7 (28.0)	
Compression	11 (44.0)	11 (44.0)	
Thyreotoxicosis	4 (16.0)	4 (16.0)	
Other	0 (0.0)	0 (0.0)	
Lymphnode dissection			
Yes	7 (28.0)	7 (28.0)	1.000
No	18 (72.0)	18 (72.0)	
Gland weight (grams)	74.0 (±61.7)	80.7 (±72.2)	0.737
Oral perioperative calcium treatment			
Yes	10 (40.0)	8 (32.0)	0.556
No	15 (60.0)	17 (68.0)	
Intravenous calcium treatment			
Yes	0 (0.0)	2 (8.0)	0.149
No	25 (100.0)	23 (92.0)	
Peroral calcium treatment			
At discharge (yes/no)	8 (32.0)/17 (68.0)	6 (24.0)/19 (76.0)	0.935
At 6 weeks follow-up (yes/no)	6 (24.0)/17 (68.0)	4 (16.0)/20 (80.0)	0.727
At 6 months follow-up (yes/no)	2 (8.0)/20 (80.0)	2 (8.0)/21 (84.0)	0.599
Total serum calcium			
Postoperative day 1	2.16 (±0.15)	2.13 (±0.16)	0.450
At 6 weeks follow-up	2.31 (±0.14)	2.27 (±0.13)	0.276
At 6 months follow-up	2.14 (±0.18)	2.26 (±0.10)	0.160
Peroral vitamin D treatment			
At discharge (yes/no)	6 (24.0)/19 (76.0)	5 (20.0)/20 (80.0)	0.733
At 6 weeks follow-up (yes/no)	4 (16.0)/19 (76.0)	3 (12.0)/21 (84.0)	0.329
At 6 months follow-up (yes/no)	1 (4.0)/21 (84.0)	2 (8.0)/22 (88.0)	0.188

*IQR* interquartile range, *GBP* gastric bypass

## Discussion

Case reports and our own experience have suggested that patients with previous GBP suffer an increased risk of severe hypocalcemia after TT [1–3], compared to patients without GBP. GBP, the most common bariatric procedure, is associated with nutritional deficiencies [22], such as low levels of 25OHD and diminished uptake of dietary calcium. Low levels of 25OHD have been shown to be a risk factor for postoperative hypocalcemia after TT and central neck dissection [8]. An increased risk of hypocalcemia after TT with previous GBP compared to TT without previous GBP is therefore plausible.

However, the hypothesis that patients with previous GBP have an increased risk of hypocalcemia after TT could not be confirmed in this large, population-based, nationwide cohort study. GBP was not a risk factor for hypocalcemia in uni- or multivariate logistic regression, and the rate of hypocalcemia was similar between previous GBP and no previous GBP in a nested case-control subset. Further, the frequency of previous GBP in patients undergoing TT was low, only 0.4 %.

The number of patients who underwent both first gastric bypass surgery and then total thyroidectomy was low in this study. However, taking into account that two nationwide registers, based on the whole Swedish population of about nine million people, were used; we consider this finding a strength rather than a weakness.

Another potential explanation for the lack of an association could be missing observations. The Swedish Obesity Register, SOReg, started in May 2007, and no bariatric procedures performed before this date could be included in our study. Hence, it is possible that our study underestimates the rate of previous GBP in patients with TT. However, this seems to be rather unlikely since only 6–18 operations/100,000 were performed 2002–2006 and 24–89 operations/100,000 were performed 2007–2014 (Fig. 1S). Furthermore, the SOReg has a coverage of almost 100 % and it is unlikely that GBPs performed after May 2007 were missed in the patients in the present study [20].

The possibility of inaccurate data must also be taken into account. If treatment for hypocalcemia is inaccurately reported to the registry, the true frequency of this complication could be underestimated. However, the SQRTPA is audited regularly and data quality has been shown to be good. Further, data on treatment for hypocalcemia would likely be missing or inaccurate at random, and it is unlikely that this would systematically bias the results.

With only 25 patients with previous GBP, the study might be underpowered to detect a true difference in the rate of hypocalcemia after TT with and without previous GBP. While we did not observe any difference, it must be emphasized that our study does not prove the absence of such a difference.

The rate of calcium and/or vitamin D treatment in the whole cohort and in the nested control group was rather high, 4.7 %. This high rate could make it difficult to detect a true increase in the risk of hypocalcemia after TT in patients with previous GBP. An explanation for the high rate of hypocalcemia in the whole cohort could be the inclusion of low-volume hospitals in the registry. Another reason for the high rate of hypocalcemia overall in this study could be the high proportion of TT for thyrotoxicosis, more than 50 %, since previous studies indicate that TT for thyrotoxicosis carries a higher risk of long-term hypocalcemia. However, when adjusting for thyrotoxicosis in the multivariate logistic regression analysis, there was no significant increased risk of hypocalcemia after TT with previous GBP.

Another explanation for the disagreement with the present study and previous case reports [1–3] could be that patients with previous GBP in the present study had sufficient levels of 25OHD, and that patients in case reports had insufficient levels of 25OHD. Unfortunately, we did not have information about levels of 25OHD.

Furthermore, the time between GBP and TT could be important. Calcium and vitamin D depletion could aggravate over time after GBP. Perhaps GBP does not increase the risk of hypocalcemia after TT during the first years postoperatively, and this might be another explanation to the lack of association between GBP and hypocalcemia after TT in our study, since the time interval was in no patient longer than 7 years.

Similar to previous studies [23–25], age, gender, indication for surgery, and lymph node dissection were risk factors for postoperative hypocalcemia in our study.

The major strengths of this study include the large number of patients with TT; the truly population-based design with registers having wide national coverage; the high external validity including 35 Swedish units, ranging from small rural county hospitals to large university hospitals; and the high data quality in terms of coverage and accuracy, with nearly 100 % coverage for obesity surgery. Further, there were low numbers of patients with missing information for oral calcium treatment at 6 weeks and 6 months [18–20].

## Conclusion

In conclusion, the results of this large observational study did not provide evidence for an increased risk for hypocalcemia after TT compared to patients without previous GBP. This study does neither support giving any extra supplementation to nor taking any extra blood tests in patients with previous GBP before TT, since their risk of hypocalcemia does not seem to markedly differ from the overall TT cohort.

## Electronic supplementary material

ESM 1 (PDF 32 kb)

## References

[1] Alfonso B, Jacobson AS, Alon EE, Via MA (2015). Previous gastric bypass surgery complicating total thyroidectomy. Ear Nose Throat J.

[2] Salinger EM, Moore JT (2010). Profound hypocalcemia after near-total thyroidectomy in a Roux-en-Y gastric bypass patient. Am Surg.

[3] Pietras SM, Holick MF (2009). Refractory hypocalcemia following near-total thyroidectomy in a patient with a prior Roux-en-Y gastric bypass. Obes Surg.

[4] Yatsuya H, Li Y, Hilawe EH (2014). Global trend in overweight and obesity and its association with cardiovascular disease incidence. Circ J.

[5] Efremidou EI, Papageorgiou MS, Liratzopoulos N, Manolas KJ (2009). The efficacy and safety of total thyroidectomy in the management of benign thyroid disease: a review of 932 cases. Can J Surg.

[6] Bhattacharyya N, Fried MP (2002). Assessment of the morbidity and complications of total thyroidectomy. Arch Otolaryngol Head Neck Surg.

[7] Yamashita H, Murakami T, Noguchi S (1999). Postoperative tetany in Graves disease: important role of vitamin D metabolites. Ann Surg.

[8] Kim WW, Chung S-H, Ban EJ (2015). Is preoperative vitamin D deficiency a risk factor for postoperative symptomatic hypocalcemia in thyroid cancer patients undergoing total thyroidectomy plus central compartment neck dissection?. Thyroid.

[9] le Roux CW, Bueter M (2014). The physiology of altered eating behaviour after Roux-en-Y gastric bypass. Exp Physiol.

[10] Shah M, Simha V, Garg A (2006). Review: long-term impact of bariatric surgery on body weight, comorbidities, and nutritional status. J Clin Endocrinol Metab.

[11] Elias E, Casselbrant A, Werling M (2014). Bone mineral density and expression of vitamin D receptor-dependent calcium uptake mechanisms in the proximal small intestine after bariatric surgery. Br J Surg.

[12] Karefylakis C, Naslund I, Edholm D (2014). Vitamin D status 10 years after primary gastric bypass: gravely high prevalence of hypovitaminosis D and raised PTH levels. Obes Surg.

[13] Liu C, Wu D, Zhang J-F (2016). Changes in bone metabolism in morbidly obese patients after bariatric surgery: a meta-analysis. Obes Surg.

[14] Lu C-W, Chang Y-K, Chang H-H (2015). Fracture risk after bariatric surgery: a 12-year nationwide cohort study. Medicine (Baltimore).

[15] Levinson R, Silverman JB, Catella JG (2013). Pharmacotherapy prevention and management of nutritional deficiencies post Roux-en-Y gastric bypass. Obes Surg.

[16] Hessman C, Fields J, Schuman E (2011). Outpatient thyroidectomy: is it a safe and reasonable option?. Am J Surg.

[17] Snyder SK, Hamid KS, Roberson CR (2010). Outpatient thyroidectomy is safe and reasonable: experience with more than 1,000 planned outpatient procedures. J Am Coll Surg.

[18] Swedish National Board of Health and Welfare: http://www.socialstyrelsen.se/english.

[19] Scandinavian Quality register for Thyroid Parathyroid and Adrenal Surgery:http://www.thyroid-parathyroidsurgerty.com/. 10.1007/s00423-006-0097-6 17103223

[20] Hedenbro JL, Naslund E, Boman L (2015). Formation of the Scandinavian obesity surgery registry, SOReg. Obes Surg.

[21] von Elm E, Altman DG, Egger M (2014). The strengthening the reporting of observational studies in epidemiology (STROBE) statement: guidelines for reporting observational studies. Int J Surg.

[22] Bal BS, Finelli FC, Shope TR, Koch TR (2012). Nutritional deficiencies after bariatric surgery. Nat Rev Endocrinol.

[23] Abboud B, Sargi Z, Akkam M, Sleilaty F (2002). Risk factors for postthyroidectomy hypocalcemia. J Am Coll Surg.

[24] Wingert DJ, Friesen SR, Iliopoulos JI (1986). Post-thyroidectomy hypocalcemia. Incidence and risk factors. Am J Surg.

[25] McHenry CR, Speroff T, Wentworth D, Murphy T (1994). Risk factors for postthyroidectomy hypocalcemia. Surgery.

