# Supplementary material for: Hypoparathyroidism after total thyroidectomy in patients with previous gastric bypass
Source: Langenbecks Arch Surg. 2016 Oct 26;402(2):273–80. doi: 10.1007/s00423-016-1517-x (PMC5346422; doi:10.1007/s00423-016-1517-x)

Figure 1S. Number of gastric bypass operations performed in Sweden 1998-2014. Data from National patient registry (1998-2014), SOReg (2007-2014) [20] and other sources

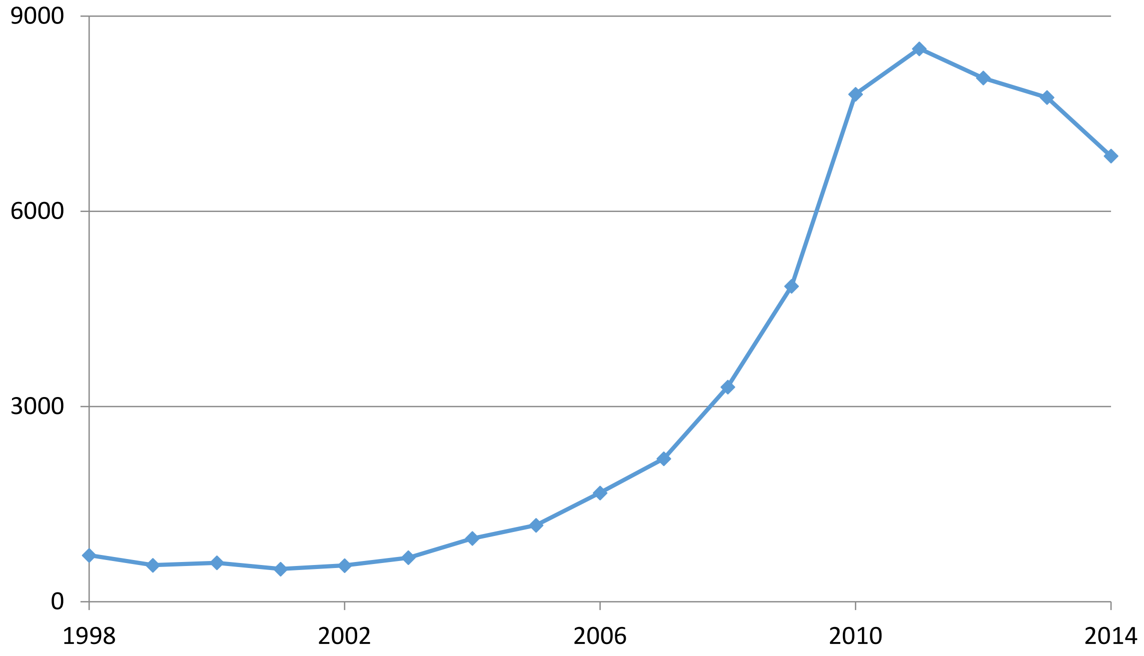

Supplement: Supplementary file 1 — (PDF 32 kb) [file 423_2016_1517_MOESM1_ESM.pdf]
